# Supplementary material for: The role of palbociclib on the alterations in CDKN2, CCNE1, E2F3, MDM2 expressions as target genes of miR-141
Source: PLoS One. 2024 Aug 8;19(8):e0306545. doi: 10.1371/journal.pone.0306545 (PMC11309483; doi:10.1371/journal.pone.0306545)
Supplement: Attachment — Submitted filename: Rebuttal letter.doc [file pone.0306545.s003.doc]

Dr Sudhir Kumar Rai

Academic Editor

PLOS ONE

Apr 15 2024

Dear Dr Kumar Rai

Re: Manuscript reference No. PONE-D-23-42371

Please find attached a revised version of our manuscript “The role of Palbociclib on the alterations in CDKN2, CCNE1, E2F3, MDM2 expressions as target genes of miR-141”, which we would like to resubmit for publication as a Research Article in PLOS One.

Your comments and those of the reviewers were highly insightful and enabled us to greatly improve the quality of our manuscript. In the following pages are our point-by-point responses to each of the comments of the reviewers as well as your own comments. Revisions in the text are shown using yellow highlight in the marked-up copy of the manuscript.

1. Please ensure that your manuscript meets PLOS ONE's style requirements, including those for file naming.

***Response:*** We thank Dr. Kumar for this valuable suggestion and we agree. We have incorporated these changes into the manuscript. We have marked all the changes with yellow highlights in the Word file labeled 'Revised Manuscript with Track Changes'.

2. We note that your Data Availability Statement is currently as follows: [ll relevant data are within the manuscript and its Supporting Information files.]

***Response:*** Thank the Editor for careful consideration, the correction was made. The data were loaded into Excel files named S1 and S2.

Response to Reviewer #1

1. Authors are suggested to re-write the manuscript in grammatically correct and in standard academic English with non-ambiguous sentence structure. The majority of the sentences are confusing and hard to understand what authors exactly want to convey.

***Response:*** Thank the Reviewer for careful consideration, The language of the manuscript was reviewed, and Some changes were made.

2- In "Results and Conclusion" section in the abstract, please write in a non-ambiguous, coherent, and clear manner. Especially regarding MDM2 gene in lines 16 and 17.

***Response:*** In this study, we investigated the changes in gene expression in two cell lines, MCF-7 and MDA-MB-231, after treatment with palbociclib. Regarding MDM2, there was a decrease in expression in the MCF-7 cell line, but no significant expression change occurred in the MDMA-MB-231 cell line.

3. In the last paragraph of "Introduction" section, after line 57, please provide brief sentences on "what you are going to do in this paper" before you move into the methods section.

***Response:*** Thank the Reviewer for careful consideration, the correction was made.

4. Authors are suggested to conduct an additional protein detection experiments, such as Western Blot to support their assumptions that are made on "Discussion section".

***Response:*** Thanks for your very insightful and detailed suggestion. We agree, but this study project is related to the master's thesis and due to financial and time shortages related to university regulations, it was not possible for us to carry out this proposed project.

5. In "Discussion" section in lines 136 and 137, please clarify using an additional sentence why Palbociclib reducing the expression of these genes are important?

***Response:*** Thank you very much for your detailed and useful comments. Reducing the expression of these genes leads to cell cycle arrest and prevents the growth of cancer cells.

6. From line 136 to 143, the remark regarding CDKN2A is clear, however, the previous conclusion with MDM2 in lines 139 and 140 is not clear. You data shows that the expression of E2F3, CCNE1 and MDM2 were reduced upon Palbociclib treatment, however, only the expression of CCNE1 was significantly reduced, thus, indicating the cell cycle arrest. Please conclude clearly about MDM2.

***Response:*** Thank the Reviewer for careful consideration, We have described this in Discussion section (line 167-169).

7. In line 165, if you can not see significant difference in mRNA level, it is unlikely that the difference can be significant at protein level. Looking for the proteins could be more confirmatory, thus authors are suggested to add protein identification experiments, such as western blot.

***Response:*** Thank you for your detailed and useful comments. According to the decrease in MDM2 gene expression and increase in CDKN2A gene expression after palbociclib treatment. By inhibiting the cyclin D-CDK4/6 D complex, Rb proteins are not phosphorylated and they are not separated from E2F transcription factors, and E2F transcription factors cannot continue their activity. Also, by increasing miR-141 gene expression, it inhibits the expression of its target genes (including E2F3) at the post-transcriptional level.

8. In "Conclusion" section from the line 196 onwards, include all the findings in brief. In line 197, Clarify "affect" means "increase" or "decrease". Clarify "Some genes"; please mention the name of the genes. Clarify "contributed to cell cycle cascade"; clarify which gene expression affects what stage of cell cycle, mentioning if it "arrests" or "promotes" the cell cycle.

***Response:*** We thank Reviewer 1 for this valuable suggestion and we agree. We modified our Discussion sections (lines 227–231)

Response to Reviewer #2

Major comments: The results for Section 2.4. Bioinformatics, are missing. The authors have mentioned that they have attempted microRNA target prediction, but no results have been provided.
Figures 2 and 3 show the same results. The authors can use one of the two figures to show the results and avoid confusion.

***Response:*** In accordance with reviewer *2*’s suggestion, We removed Figure 3 and added Figure 1 as results for Section 2.4. Bioinformatics.

minor comments:

Line 62: Correct ‘CO2’. 2 should be subscript.
Line 65,76,76: Correct the temperature notation to ‘°C’.
Figure 2: There is no pie chart. Please correct.
Section4. Correct ‘discussion’ to ‘Discussion’
Use uniform notation for Figures throughout the manuscript.

***Response:*** Thank Reviewer 2 for careful consideration, the correction was made.

We hope that the revisions in the manuscript and our accompanying responses will be sufficient to make our manuscript suitable for publication in PLOS ONE.

We shall look forward to hearing from you at your earliest convenience.

Yours sincerely,

Dr Arshad Hosseini

Head and faculty member of Medical Biotechnology Department, School of Allied Medical Sciences, Iran University of Medical Sciences, Tehran, I.R. Iran, zip code:1449614535

Fax: +982188622533

E-mail: hoseini.a@iums.ac.ir

**Responses to the comments of Reviewer #1**

1. *[Insert Comment here: copy and paste directly]*

*Response: [Insert response here in italics]*

2. *[Insert Comment here: copy and paste directly]*

*Response: [Insert response here in italics]*

**Responses to the comments of Reviewer #2**

1. *[Insert Comment here: copy and paste directly]*

*Response: [Insert response here in italics]*

2. *[Insert Comment here: copy and paste directly]*

*Response: [Insert response here in italics]*
