# Supplementary material for: The role of palbociclib on the alterations in CDKN2, CCNE1, E2F3, MDM2 expressions as target genes of miR-141
Source: PLoS One. 2024 Aug 8;19(8):e0306545. doi: 10.1371/journal.pone.0306545 (PMC11309483; doi:10.1371/journal.pone.0306545)
Supplement: Attachment — Submitted filename: Rebuttal letter.doc [file pone.0306545.s004.doc]

Dr Sudhir Kumar Rai

Academic Editor

PLOS ONE

Jun 13 2024

Dear Dr Kumar Rai

Re: Manuscript reference No. PONE-D-23-42371

Please find attached a revised version of our manuscript “The role of Palbociclib on the alterations in CDKN2, CCNE1, E2F3, MDM2 expressions as target genes of miR-141”, which we would like to resubmit for publication as a Research Article in PLOS One.

Your comments and those of the reviewers were highly insightful and enabled us to greatly improve the quality of our manuscript. In the following pages are our point-by-point responses to each of the comments of the reviewers as well as your own comments. Revisions in the text are shown using yellow highlight in the marked-up copy of the manuscript.

Please review your reference list to ensure that it is complete and correct. If you have cited papers that have been retracted, please include the rationale for doing so in the manuscript text, or remove these references and replace them with relevant current references. Any changes to the reference list should be mentioned in the rebuttal letter that accompanies your revised manuscript. If you need to cite a retracted article, indicate the article’s retracted status in the References list and also include a citation and full reference for the retraction notice.

***Response:*** Following your suggestion, We removed reference 3 and checked the reference list to ensure that it is complete and correct.

Response to Reviewer #1

1.Regarding the previous comment, “In the Results and Conclusion section in the abstract, please write in a non-ambiguous, coherent, and clear manner. Especially regarding MDM2 gene in lines 16 and 17”, I understand that palbociclib treatment reduced the expression of MDM2 and CCNE1 expression in MCF-7 cells. However, the first sentence in the results and conclusion section in your abstract is still ambiguous. Please correct the first sentence and avoid repetition in the latter sentences in your manuscript.

***Response:*** Thank the Reviewer for careful consideration, the correction was made.

2. Academic standard English is still lacking and ambiguity is still present, please revise the manuscript thoroughly.

***Response:*** Thank the Reviewer for careful consideration, The manuscript was revised and changes were made.

We hope that the revisions in the manuscript and our accompanying responses will be sufficient to make our manuscript suitable for publication in PLOS ONE.

We shall look forward to hearing from you at your earliest convenience.

Yours sincerely,

Dr Arshad Hosseini

Head and faculty member of Medical Biotechnology Department, School of Allied Medical Sciences, Iran University of Medical Sciences, Tehran, I.R. Iran, zip code:1449614535

Fax: +982188622533

E-mail: hoseini.a@iums.ac.ir

**Responses to the comments of Reviewer #1**

1. *[Insert Comment here: copy and paste directly]*

*Response: [Insert response here in italics]*

2. *[Insert Comment here: copy and paste directly]*

*Response: [Insert response here in italics]*

**Responses to the comments of Reviewer #2**

1. *[Insert Comment here: copy and paste directly]*

*Response: [Insert response here in italics]*

2. *[Insert Comment here: copy and paste directly]*

*Response: [Insert response here in italics]*
